# A Brief Introduction to the Multidimensional Intercultural Training Acculturation Model (MITA) for Middle Eastern Adolescent Refugees

**DOI:** 10.3390/ijerph15071516

**Published:** 2018-07-18

**Authors:** Atefeh Fathi, Usama El-Awad, Tilman Reinelt, Franz Petermann

**Affiliations:** Zentrum für Klinische Psychologie und Rehabilitation, Universität Bremen, Grazer Str. 6, 28359 Bremen, Germany; elawad@uni-bremen.de (U.E.-A.); reinelt@uni-bremen.de (T.R.); fpeterm@uni-bremen.de (F.P.)

**Keywords:** multidimensional intercultural training acculturation model (MITA), intercultural competence, traumatic events, mental health, Middle Eastern refugee adolescents

## Abstract

The large number of adolescent refugees around the world constitutes a great challenge for societies. However, current models of acculturation have been developed for migrants, but not specifically for adolescent refugees. Crucial factors to describe adolescent refugee acculturation, such as intentions to return to their homeland, especially with respect to adolescent refugees with temporary residency and experiences of potentially traumatic events, are missing. Hence, the Multidimensional Intercultural Training Acculturation (MITA) model is introduced. The model proposes that two major concerns for adolescent refugees, which are socio-cultural adjustment and mental health, are predicted by intercultural and social–emotional competence, intentions to return to their homeland, and experiences of traumatic events. Moreover, the effects of three modes of acculturation are also proposed in the model. It is expected that these variables mediate the effects of intercultural competence, social–emotional competence, intentions to return to the homeland, and experiences of traumatic events on socio-cultural adjustment as well as mental health. Finally, it is also expected that in-group social support and out-group social support moderate the direct connection between the experiences of traumatic events and mental health.

## 1. Introduction

According to the United Nations Refugee Agency’ reports, the number of people around the world forced from home because of war, human rights violations, persecution, or generalized violence and who need resettlement increased from 33.9 million in 1997 to 65.6 million in 2017 [1]. Altogether, more than half (55 percent) of all refugees worldwide come from Middle Eastern countries. Syrians are considered to be the largest resettled population, with 12 million refugees at the end of 2016. Afghans are the second largest group, followed by Iraqis [1]. Among European countries, Germany has received the largest number of refugees, at about 746,649 in 2017. Nearly one-third of these refugees were under the age of 18 [2]. The most common Middle Eastern country of origin for refugee adolescents and children was Afghanistan, followed by Syria and Iraq [1]. Some of these adolescent refugees may be psychologically vulnerable due to the experience of potentially traumatic events such as experiencing separation from their parents or close family members, or even their deaths. In addition, they may be confronted with cultural identity confusion during the process of resettlement. This complicated mixture of experiences may make their process of acculturation especially stressful [3].

Acculturation is defined as a cultural modification of individuals by adapting to another culture. In other words, acculturation is the process of cultural and psychological changes that occur because of the interaction between immigrants and members of the host culture [4]. At the cultural level, both the host and origin cultures usually have some priorities or targets for immigrants to attain (e.g., form of communication, eating habits, and dressing style) which lead them to choose different types of acculturation strategies [4]. At the psychological level that comprises cultural shedding, culture learning, and cultural conflict, behavioral rules change, which is usually non-problematic. Although cultural shedding and cultural learning may be selective, accidental, or deliberate, they productively allow the individual to adapt to the society of settlement [4]. However, cultural conflicts are expected to be problematic and affect refugees’ social behaviors and jeopardize their mental health [5]. This is especially relevant for adolescent refugees, as their identity development is positively related with some crucial factors such as belonging to a peer group and good social relationships based on mutual respect and acceptance [6,7]. Therefore, adolescent refugees need long-term and comprehensive solutions to rebuild their fractured identities [8]. For example, psychological and social supports and offers from youth welfare services are able to reduce many difficulties and prepare valuable comprehensive care services [9,10]. Moreover, some multimodal psychosocial supports and school-based programs [11] as well as some specific training programs for adolescent refugees that concentrate both on changing the environment and changing the adolescent refugees’ skill sets (e.g., emotional skills such as emotional regulation, social skills such as conversational skills, and behavioral skills such as assertiveness and empathy) might be especially promising [12,13].

However, despite the overwhelming numbers of adolescent refugees, all current acculturation models are specific to immigrants or adult refugees (e.g., the Multidimensional Individual Differences Acculturation model: MIDA [14], the Relative Acculturation Extended Model: RAEM [15], and Rudmin’s model of acculturation as second-culture acquisition [16]). Therefore, a new acculturation model specific to adolescent refugees is needed to improve the knowledge on this population, especially on those from the Middle East living in European countries, and more importantly to evaluate their social behaviors and mental health. The model we propose, builds on and combines elements from three different models of acculturation: (1) Berry’s bi-dimensional model of acculturation [17], (2) the Multidimensional Individual Differences Acculturation model [14], and (3) Rudmin’s [16] acculturative learning model.

### 1.1. Bi-Dimensional Model of Acculturation 

According to Berry’s bi-dimensional model of acculturation [17], there are two principal factors in estimating acculturation: (1) retention of the heritage culture, and (2) attainment of the new culture. These two principal factors result in four acculturation strategies which are as follows: integration (i.e., retention of the heritage culture as well as attainment of the new culture), separation (i.e., retention of the heritage culture but no attainment of the new one), assimilation (i.e., abandonment of the heritage culture and adoption of the new culture), and marginalization (i.e., abandonment of the heritage culture as well as failure to adapt to the new culture) [18]. Inconsistencies and conflicts between these different acculturation strategies can lead to psychological difficulties [18]. Hence, acculturative stress may be observed when acculturation experiences result in psychological problems for refugees. Berry discussed that although bi-cultural (integrated) individuals must be more under pressure from both the heritage and host culture communities, they generally have a better psychological adaptation [4] and if their acculturation strategy does not lead to success, they are sufficiently flexible to modify it [18]. Separated individuals have more contact with people of their heritage culture and receive more support from them. However, they report more pressure from the host society to adapt to the receiving culture and are most likely to face discrimination from members of the host society [19]. Assimilated people are characterized by low ethnic identity as well as a high national identity of the host country. Although they may have more contact with people of the host country and report fewer experiences of discrimination, they suffer from low support from their family and the members of their ethnic groups. Marginalized individuals demonstrate “cultural identity confusion” [18,20]. They show a higher degree of lack of interpersonal trust, self-assurance, and neuroticism [21]. Berry [4] noted that the idea of marginality was synonymous with the concept of deculturation, which is broadly used as the process where aspects of one culture are lost after contact with another one. Individuals with a marginalization strategy are highly averted from their original culture as well as the culture of the dominant group. Marginalized people profoundly suffer from acculturative stress, which is the psychological effect of adaptation to a new culture [22,23]. Acculturative stress is defined as a reduction in the psychological health of immigrants and refugees [24].

### 1.2. The Acculturative Learning Model

Rudmin discussed that the concentration on acculturative stress neglects the motivation to acculturate [25]. For example, sojourners, skilled workers, missionaries, business agents, and students show that it is achievable to acculturate purely because of some motivational reasons, even if the attitudes to the host culture are not entirely positive or are even negative. Moreover, acculturation as a process that requires resources such as mental energy, money, time, and social capital, and in some cases the risks of negative consequences, may necessitate some cost–benefit estimations [16]. Rudmin’s model contains three steps, including acculturative motivation, acculturative learning, and alterations in the individuals. Rudmin’s model [16] of acculturation as second-culture acquisition explains that the motivation to acculturate leads to acculturative learning, which may bring about some changes in individuals (e.g., such as communication style, lifestyle, values, moral codes, clothing, the way of thinking, social activities). The alterations that arise from acculturative learning may be outcomes of the family situation, successes, failures, political activities, and creativity as well as discrimination. Motivation to acculturate can involve: (1) cultural attitudes, (2) ethnic identity, (3) reacting to stress, and (4) utility, involving the risks and costs of second-culture acquisition [16]. This model claims that the four main methods of acculturative learning are: (1) information about the new culture, (2) instructions, (3) imitation of new culture behaviors, and (4) mentoring by persons competent in the host culture and caring enough about the acculturating person to be individually supportive. Finally, in Rudmin’s model, perceptions about the socioeconomic status as well as discrimination are considered as control variables. They can influence how an individual is learning a second culture. However, these factors alone do not result in acculturation. Therefore, these variables are not considered as learning a second culture [26].

### 1.3. The Multidimensional Individual Differences Acculturation Model 

The Multidimensional Individual Difference Acculturation (MIDA) model was initially developed by Safdar et al. [14] using first-generation Iranian immigrants living in Canada. This model was empirically examined with immigrants of diverse ethnic origins living in urban and rural areas in Canada [14], Iranian immigrants living in the Netherlands, the United Kingdom, and the United States [27], and Indian and Russian immigrants living in Canada [28]. Primarily, the MIDA model suggested that acculturation attitudes and coping resources are more essential predictors of psycho-physical health outcomes than demographic variables [14]. In the MIDA model, three factors are predictors of acculturation attitudes and adaptation outcomes. These predictor variables are: (1) psychosocial resources, (2) co-national connectedness, and (3) hassles. Psycho-social resources contain three components: resilience, cultural competence, and out-group social support (social support from the host society). Resilience focuses on the existence of positive psychological functioning. Cultural competence refers to immigrants’ communication abilities in the host society and their cultural efficacy. Out-group social support refers to the social support from members of the host society [14]. Co-national connectedness is the second predictive factor that consists of three components: ethnic identity, family allocentrism, and perceived family and in-group social support. Ethnic identity focuses on the identification with the heritage group. Family allocentrism refers to the quality of family ties and relationships [14]. In-group social support refers to the perceived support from family and in-group members. In the MIDA model, distinguishing between in-group social support and out-group social support is very important to predict the immigrants’ psychological well-being. Hassles allude to chronic irritants that individuals frequently face, such as time pressure, financial difficulties, arguing with friends/family members, and being overburdened with responsibilities [14]. The outcome variables in this model are in-group behavior from Co-national connectedness, out-group behavior from psycho-social resources, and psycho-physical distress from hassles [27,28]. Types of acculturation strategies [17] are also evaluated in MIDA model, which are attitudes toward old culture maintenance (separation) and new culture acquisition (assimilation). These types of acculturation strategies are considered as mediating variables in connecting the MIDA model’s psychological constructs to the output [14]. In this mediating situation, co-national connectedness predicts separation or old culture maintenance and separation predicts in-group contact [27,28]. Hassles predict separation as well as psychophysical distress. Psycho-social resources predict assimilation or new culture acquisition and assimilation predicts out-group contact [14,27].

### 1.4. Limitation of the Acculturation Models Regarding Adolescent Refugees

Traditionally, Berry’s model of acculturation has been the dominant paradigm in acculturation studies. However, a common criticism of Berry’s acculturation model is that it has primarily concentrated on the acculturation of permanent adult migrants and has been mainly employed with voluntary adult refugee and immigrant samples. Therefore, some critical factors such as intercultural competence as well as acculturative learning [25], which can play essential roles in acculturation orientation (especially for adolescent refugees with temporary residency), have been ignored.

Although some essential factors related to acculturative learning are included in Rudmin’s model [16], there are still some deficiencies that should be taken into consideration. First of all, information has rarely been scrutinized for its effectiveness as a procedure of second-culture learning, including informal information found in movies, novels, and music [29,30]. Furthermore, instructions, as intercultural training, have commonly concentrated on preparing students, skilled workers, and sojourners [31]. Thus, proper information about the host culture and the concept of culture itself, as well as intercultural training, are needed to be applied more broadly (e.g., most important for adolescent refugees who come from completely different cultures and ethnicities). Finally, considering the utility decision that works by the decision between the costs and the benefits [16], intentions to return to the homeland should be taken into consideration. Intentions to return to the homeland can affect the motivation of adolescent refugees to establish and to maintain their intercultural relationships. Therefore, a new model for adolescent refugees needs to fill the gap by applying intentions to return to the homeland as well as intercultural competence as factors, which mainly contain motivation to acculturate, cultural knowledge, and intercultural training as their latent components.

In addition, some critical factors, including social–emotional competencies and experiences of traumatic events, which can affect adolescent refugees’ mental health and socio-cultural adjustment as well as acculturation orientations, are mainly neglected in the MIDA model. Because of the nature of its variables, the MIDA model is essentially culture non-specific. Moreover, its outcome variables, including out-group/in-group contacts as well as psychological/physical health, are fundamental issues in multicultural societies as well as common goals of all immigrants. However, this model has been applied mostly to educated immigrants or adult individuals with immigration backgrounds [14,27,28]. Adolescent refugees with temporary residency, lower education, and insufficient social experiences are not considered.

In general, some crucial factors, such as intentions to return to the homeland (especially for adolescent refugees with temporary residency and experiences of potentially traumatic events which make the adolescent refugees psychologically fragile and vulnerable), can differentiate the acculturation models specific to refugee populations from the models of acculturation specific to immigrants. To overcome all these limitations, we propose the Multidimensional Intercultural Training Acculturation Model (MITA).

## 2. The Multidimensional Intercultural Training Acculturation Model (MITA)

The MITA model has derived some elements from three different models of acculturation, including three modes of acculturation (assimilation, separation, and integration) from Berry’s bi-dimensional model of acculturation [17], out-group social support, in-group social support, and mental health from Multidimensional Individual Differences Acculturation model (MIDA) [14], and intercultural competence as acculturative learning from Rudmin’s model [16] as second-culture acquisition. The MITA model has also derived some elements from different approaches to acculturation research, including Berry’s [4,17,18] two dimensions of adoption of the host culture and maintenance of the heritage culture and the difference between socio-cultural adjustment and psychological adjustment [32]. The MITA model has added some new elements, including intentions to return to the homeland, the experience of traumatic events, and socio-cultural adjustment. Furthermore, The MITA model is supported by a number of theoretical approaches within clinical, social, and cross-cultural psychology, including acculturation attitudes [17] and Hammer et al.’s [33] theoretical framework of Intercultural Communicating Competence (ICC) for conceptualizing intercultural competence. To conceptualize social–emotional competence, empathy from prosocial behaviors [34], and among appraisal theories, the emotion regulation strategies of Gross [35], including: (1) situation selection, (2) situation modification, (3) attentional deployment, (4) cognitive change, and (5) response-focused strategies, are selected. In the MITA model, four factors are predictors of mental health and socio-cultural adjustment. These predictor variables are (1) intercultural competence, (2) social–emotional competence, (3) intentions to return to the homeland, and (4) experiences of traumatic events.

The outcome variables in this model are socio-cultural adjustment from intercultural competence, social–emotional competence, and intentions to return to the homeland and mental health from social–emotional competence, as well as the experience of traumatic events.

Modes of acculturation [17] including integration, separation, and assimilation, are also proposed in the MITA model. These modes of acculturation are considered as mediating variables in connecting the MITA model’s psychological constructs to the output variables. In this mediating situation, intentions to return to the homeland predict separation and separation predicts socio-cultural adjustment as well as mental health. Intercultural competence predicts integration and integration predicts socio-cultural adjustment as well as mental health. Social–emotional competence predicts assimilation and assimilation predicts socio-cultural adjustment. Experience of traumatic events predicts separation and separation predicts socio-cultural adjustment.

**Figure 1:**
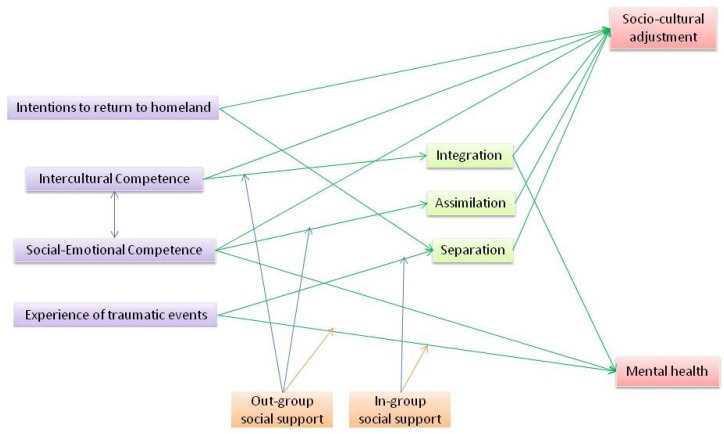
Multidimensional Intercultural Training Acculturation Model (MITA).

Moreover, the direct connection between intercultural and social–emotional competence/experiences of traumatic events, and the three modes of acculturation is influenced by two variables, including out-group social support and in-group social support. It is expected that these two variables would play some moderated mediating roles in linking intercultural competence, social–emotional competence, and experiences of traumatic events to modes of acculturation. In this moderated mediating situation, out-group social support affects the connection between intercultural and social–emotional competence and integration as well as assimilation. In-group social support affects the connection between the experience of traumatic events and assimilation. Finally, in-group social support and out-group social support are considered as moderating variables in connecting the experience of traumatic events to mental health. The connections among predictor variables, output variables, mediating variables, moderating variables, and moderated mediating variables are shown in Figure 1.

In sections to follow, each of these variables mentioned above in the MITA model is discussed further.

### 2.1. Intercultural Competence 

The term intercultural competence describes the capability to appropriately and effectively carry out social interactions and communicate with people from various cultures [32]. The refugees’ ability to communicate can facilitate all other aspects of adaptation and adjustment in the host culture. Therefore, intercultural competence may be considered as the fundamental process as well as the main outcome of the acculturation process [32]. According to Berry [17], there are three phases of acculturation processes, including contact, conflict, and adaptation. In these phases contact is a core concept for acculturation. In the contact phase, communication is remarkably important and needs to be constructive and without failures and misunderstandings as much as it is possible [17]. According to Hammer et al., [33] communication in the contact phase to acculturation is viewed as one of the common processes of intercultural competence. Based on this, there is an increase in intercultural sensitivity to cultural differences during acculturation [33]. Intercultural sensitivity comprises the ability to experience and distinguish pertinent cultural differences and is considered a focal competence for intercultural communication [36]. Hence, to understand acculturation successfully, the interactional context in which this process takes place needs to be clarified [37]. As most of the current adolescent refugees in the Western countries are from non-European backgrounds [38], the term culture has become an important element to understand the process of acculturation. As acculturation refers to cultural change, it is a requisite to determine how the term culture is described [39]. According to Shore [40], culture refers to shared understandings, meanings, or referents kept by a specific group of people. As noted by Rudmin [25], the similarity between the host culture and the heritage culture can clarify how much acculturation is needed to adapt to the host culture. Therefore, research on the concept of cultural awareness, cultural similarities, and differences can play a constructive role in socio-cultural adjustment. However, different cultural groups can bring up racial [41] as well as ethnic stereotypes [42], which might result in stereotype threat. Stereotype threat emerges when a person is in a situation having a fear of behaving in a way that would accidentally confirm a negative stereotype [43]. It should be mentioned that stereotype threat can lead to spotlight anxiety which may bring about vigilant worry and this experience of emotional distress can undermine social and emotional performance [43]. Therefore, it is expected that increasing knowledge about different types of stereotypes can be helpful to reduce its threat to social and emotional and consequently, to improve the socio-cultural adjustment. Hence, based on these explanations, in the MITA model, intercultural competence contains three components as follows: (1) cultural awareness, (2) knowledge about cultural similarities and differences, and (3) knowledge about stereotypes. In the MITA model, intercultural competence predicts socio-cultural adjustment. Moreover, the connection between intercultural competence and mental health is mediated by integration.

### 2.2. Social–Emotional Competence 

Social–emotional competence is defined as the ability to interact with people, regulate emotions and behaviors properly, solve problems reasonably, and communicate impressively. In the MITA model, social–emotional competence contains two components as follows: (1) emotion regulation and (2) empathy.

Emotion regulation is the ability to react to different experiences with the range of emotions in a way that is socially acceptable as well as the ability to postpone impulsive reactions when needed [44]. According to Gross [45], improving one’s ability for emotion regulation will lead to a greater ability to respond to emotional experiences appropriately. Several studies have confirmed the link between emotion dysregulation and impaired anger management ability [46,47,48]. Moreover, there is some experimental evidence that the execution of adaptive emotion regulation strategies can significantly diminish rage and aggression [49,50]. Although studies so far mostly have failed to confirm the link between post-migration living difficulties and emotion dysregulation, it might be the case that these daily living difficulties also affect refugees’ capability to regulate their emotions appropriately. Consequently, impaired emotion regulation might act as a mechanism for empowering the connection between refugees’ experiences and mental health outcomes [9,51]. This means that the experience of extreme emotional distress in connection with trauma and post-migration living difficulties might force the refugees to use dysfunctional emotion regulation strategies and the lack of access to functional strategies may thus empower the association between post-migration living difficulties and psychological difficulties [9]. Therefore, in the proposed MITA model, emotion regulation is added as one of the components of the social–emotional competence. Another component of social–emotional competence that is strongly linked to an appropriate emotion regulation is empathy [52]. According to Hammer et al. [33], empathy enables a person’s adaptation to differences in the course of intercultural sensitivity. According to Bar-Tal [34], empathy is a fundamental motivating factor for prosocial behavior, which is defined as a social behavior that benefits society or other individuals as a whole and emotion regulation can moderate the degree to which empathy is associated with prosocial behavior [53]. Several studies revealed that people with better abilities to regulate their emotions properly show a greater increase in prosocial behavior and empathy [54,55]. Hence, because of this significant positive association between empathy and emotion regulation, empathy is also added as one of the components of social–emotional competence. Therefore, in MITA model, social–emotional competence predicts mental health as well as socio-cultural adjustment. Moreover, it is also expected that assimilation mediates the connection between social–emotional competence and socio-cultural adjustment. 

### 2.3. Intentions to Return to the Homeland

According to Lu [55], because of different reasons (including economic, social and political restrictions), not all refugees who intend to return to their homeland necessarily go back. Refugees’ intentions to “return” can give us a great understanding of their future plans as well as their opinions about their experiences in the host countries. This information may help researchers and mental health practitioners in the domain to develop training programs which can enhance refugees’ skills and experiences in areas that they need the most [56,57]. Intentions to return to the homeland can affect the desire to acquire knowledge of the host culture, to develop connections with members of the host society, and to learn about the host country’s social and cultural environments which all considered as motivation to acculturate [58]. If acculturation is considered to be the process of acquiring knowledge of a host culture [17], intentions to return to the homeland can affect the willingness to take part in this process [59]. Its impact on the adolescent refugees, however, is still poorly investigated. Therefore, in the MITA model, intention to return to homeland is added as a predictor of socio-cultural adjustment. In the mediating situation, intentions to return to homeland predict separation and separation predicts socio-cultural adjustment as well as mental health.

### 2.4. Experience of Traumatic Events

Exposure to violence (both pre- and post-migration) as well as parents or close family members’ exposure to violence is the most studied risk factor for mental health difficulties for adolescent refugees [60]. In a study of adolescent Iraqi refugees, the experience of traumatic events predicted mental health difficulties [61]. In addition, the adolescents of non-tortured refugee families from different Middle Eastern countries appeared to have healthier mental states than the adolescent refugees from Iraq [62]. Despite the important effects of the experience of potentially traumatic events on adolescent refugees’ mental health [51,63], none of the models of acculturation have considered it as an important factor. Therefore, in the MITA model, the experience of traumatic events is added to predict the mental health of adolescent refugees. In-group/out-group social support also moderates the connection between experiences of traumatic events and mental health. Moreover, in the MITA model, the connection between experiences of traumatic events and socio-cultural adjustment is mediating by separation.

### 2.5. Modes of Acculturation 

The modes of acculturation, which have been identified by Berry [17] as separation, assimilation, integration, and marginalization, refer to ways of adapting to the new culture. However, as the acculturation mode of marginalization can be considered as a psychopathological process rather than an acculturation mode [14], only separation, assimilation, and integration are included in the model. These three modes of acculturation are considered as mediating variables in connecting the MITA model’s psychological constructs to the output variables. In this mediating situation, intentions to return to homeland predict separation and separation predicts socio-cultural adjustment as well as mental health. Intercultural competence predicts integration and integration predicts socio-cultural adjustment as well as mental health. Social–emotional competence predicts assimilation and assimilation predicts socio-cultural adjustment. Experience of traumatic events predicts separation and separation predicts socio-cultural adjustment.

### 2.6. Mental Health 

Psychological health during the acculturation process is a common goal of refugees and host countries [64]. Refugees, especially adolescent refugees, are of particular interest, as they are arguably the most at-risk group for mental disorders among all immigrants [65]. Adolescent refugees might experience acculturative stress when being confronted with different languages, dressing styles, or eating habits [66]. Hence, adolescent refugees often suffer from mental disorders such as depression, anxiety, and in some cases psychosis [52,67]. There are numerous studies examining the mental health issues experienced by refugee children and adolescents, and all of them have come to an agreement that the process of the resettlement can make them extremely vulnerable and fragile because of their underdevelopment of psycho-social and coping skills [68], inability to handle and manage certain life events, dependency, and incomplete biological development [62]. As almost half the world’s refugee population includes children and adolescents [2], to clarify the process of acculturation, any model of acculturation specific to adolescent refugees should ultimately be involved with the state of psychological health. Therefore, mental health as an outcome variable is added to the MITA model.

### 2.7. Socio-Cultural Adjustment

In general, adjustment refers to the changes which occur in oneself (such as thoughts, emotions, actions, strategies) and the interaction that facilitates the process of adaptation [17,18]. Socio-cultural adjustment is a behavioral as well as a practical feature of adapting to a host culture. Socio-cultural adjustment can be defined as an ability to fit in properly or interact with members of the host culture, effectively [32].

During the process of acculturation, socio-cultural adjustment can be challenging. Some factors, such as cultural distance, quantity and quality of social interactions with both host and heritage cultures, acculturation attitudes toward the host society, intercultural training, emotional skills (e.g., emotional regulation), social skills (conversational skills), behavioral skills (e.g., assertiveness, empathy), previous cross-cultural experiences [69], interest in the host culture’s national events, and sharing interest and friendships with members of the host culture [32,69] were found to be combined with socio-cultural adjustment. Therefore, in the MITA model, the socio-cultural adjustment is added as an outcome variable which is predicted from intercultural competence, social–emotional competence and intentions to return to the homeland. In the mediating situation, the connection between intentions to return to the homeland and socio-cultural adjustment is mediated by separation. Integration mediates the connection between intercultural competence and socio-cultural adjustment. Social–emotional competence predicts assimilation and assimilation predicts socio-cultural adjustment. Experience of traumatic events predicts separation and separation predicts socio-cultural adjustment.

### 2.8. Out-Group Social Support/In-Group Social Support

In general, social support may have a remarkable influence on the mental state. A high amount of available social support in the new culture and the nature of the host society can have a considerable effect on minimizing acculturative stress and empower individuals to handle the difficulties of living in a diverse environment [70,71]. Availability of support systems is an important post-migration factor that can facilitate successful adaptation for adolescent refugees even when they have gone through traumatic experiences [72,73,74]. Conversely, a lack of social support can be related to a poor psychological adaptation [75,76]. With respect to the positive effect of social support, the connection with the close ethnic groups has been found to have a positive effect on mental health in children and adolescents [77,78]. Furnham and Bochner [79] reported an opposite relationship between the prevalence of mental disorders and the perceived social support from the members of the heritage culture. The protective nature of positive peer relationships, as well as good parental mental health, have been highlighted in various studies [74,80,81,82]. Adolescent refugees in close contact with their nuclear families and close family members showed less emotional distress and greater adjustment than those who survived the refugee process alone [82].

Therefore, in the proposed model, out-group and in-group social support are added as moderated mediating variables to affect the connection between intercultural and social–emotional competence/experiences of traumatic events and modes of acculturation. In this moderated mediating situation, out-group social support affects the connection between intercultural and social–emotional competence and integration as well as assimilation. In-group social support affects the connection between the experience of traumatic events and assimilation. Moreover, in-group social support and out-group social support are also considered as moderating variables in connecting the experience of traumatic events to mental health.

## 3. Discussion

Overall, the MITA model proposes that two major concerns of adolescent refugees, including socio-cultural adjustment and mental health, are predicted by intercultural as well as social–emotional competence, intentions to return to the homeland, and experiences of traumatic events. In addition to the direct influence of intercultural competence, social–emotional competence, intentions to return to the homeland, and experiences of traumatic events on mental health and socio-cultural adjustment, the effects of three modes of acculturation (integration, separation, and assimilation) are also proposed in the model. It is expected that these variables mediate the effects of intercultural competence, social–emotional competence, intentions to return to the homeland, and experiences of traumatic events on socio-cultural adjustment as well as mental health.

Moreover, the direct connection between intercultural and social–emotional competence/experiences of traumatic events and the three modes of acculturation is influenced by two variables, including out-group social support and in-group social support. It is expected that these two variables would play some moderated mediating roles in linking intercultural competence, social–emotional competence, and experiences of traumatic events to modes of acculturation. Finally, it is also expected that in-group social support and out-group social support moderate the direct connection between experiences of traumatic events and mental health. This proposed model can help researchers in the domain to understand adolescent refugees’ behaviors, lifestyle, and beliefs in different ethnicities by evaluating aspects of intercultural and social–emotional competence, which are not addressed in current acculturation models. For instance, considering intentions to return to the homeland, our proposed model can clarify the reasons why adolescent refugees with different ethnicities and socio-cultural characteristics may select different acculturation strategies, even when they live in the same society. Moreover, using intercultural and social–emotional competence, the model can explain why adolescent refugees from the same countries and socio-cultural backgrounds or even same families may choose different acculturation strategies. At the clinical level, this model highlights socio-cultural reasons behind some mental health problems among adolescent refugees. The relationship between acculturation strategies and the social traditions in the host society can explain why adolescent refugees get involved with psychological problems. Besides, for intervention purposes, it can help clinicians to apply some special training programs involving the socio-cultural characteristics of adolescent refugees (e.g., group intercultural competence training [56]). Despite the theoretical evidence, a great deal of applied and basic research is still required to test the fundamental assumptions of the MITA model.

## 4. Limitations of the MITA model

Because of the nature of this concept paper, the proposed MITA model has not been tested yet, and therefore, its factors should be taken with caution and viewed as hypotheses. Moreover, time is not explicitly considered in the proposed model: levels of intercultural and social–emotional competence, intentions to return to the homeland, and their relations with the modes of acculturation as well as socio-cultural adjustment and mental health may vary with the length of residency in the host country. Finally, the levels of connections among the variables in the proposed model are mostly defined according to monocultural societies (or societies that promote monoculturalism). Therefore, the relations among the variables might differ in multicultural societies. 

## 5. Implications for Future Research

In the future, the proposed model should be tested and validated empirically. Furthermore, the model should be tested using samples of refugee adolescents from different ethnicities (e.g., such as those of African, Indian, Asian, and Pakistani origin). This would be an important step in assuring the contribution of the present model to acculturation processes and the intercultural and social–emotional competence of various ethnicities. Finally, it is essential to test the MITA model and especially its proposed moderators and mediators over time in various countries with different approaches to culture, for example with Canadian multiculturalism and German monoculturalism.

## 6. Conclusions

To the best of our knowledge, no research study has been accomplished on establishing an acculturation model specific to adolescent refugees connecting social–emotional and intercultural competence, the experience of traumatic events, and intentions to return to the homeland to mental health as a psychological outcome, and socio-cultural adjustment as a behavioral outcome using acculturation attitudes as mediating variables and in-group/out-group social support as moderating as well as moderated mediating variables. In this sense, the MITA model is best regarded as exploratory, with further investigation required.
